# Shedding light on light pollution

**DOI:** 10.1016/j.lanepe.2023.100710

**Published:** 2023-08-01

**Authors:** The Lancet Regional Health – Europe

In July, countries of the WHO European Region met for the 7th Ministerial Conference on Environment and Health in Hungary. A commitment was made to prioritise wide-ranging action to address health challenges related to climate change and environmental pollution. However, light pollution—excessive or misdirected artificial light at night (ALAN)—which impacts at least 80% of the world's population and more than 99% of Europeans, was not part of the discussion, and therefore no specific actions were proposed to tackle it. As traditional lighting technology is replaced by light emitting diodes (LEDs) in an attempt to reduce energy consumption, carbon emissions, and energy costs, the artificially lit surface of the Earth at night continues to increase in brightness and extent. This increased use of LEDs as a result of their lower cost has increased light pollution by 49% between 1992 and 2017. Thus, it is imperative that light pollution is viewed and treated with the same importance as other types of environmental pollution.

Light pollution can come from many different sources, such as unintentional illumination of outdoor spaces, excessive lighting that results in undesirable brightness, and the use of light emitting short optical length radiation, such as blue light from devices that we use indiscriminately (e.g., smartphones). Although light pollution is pervasive, it was not until 2020, when the EU Biodiversity Strategy for 2030: Bringing nature back into our lives was launched, that light pollution was finally recognised as an environmental problem. This strategy called on the European Commission and Member States to address light and noise pollution and to propose guidelines to reduce the use of ALAN and set an ambitious reduction target for 2030. However, to date, no ambitious targets have been set and no stringent guidelines have been applied.

Similar to noise pollution, light pollution is neglected due to lack of awareness that ALAN is a pollutant that negatively affects health, wildlife, marine life, and climate. Furthermore, international institutions and public policy makers have not included ALAN among their priorities for tackling environmental pollution. Some European countries (Slovenia, Croatia, Czech Republic, and France) have a progressive national legal framework to restrict ALAN, but without a uniform approach across Europe and infrastructure to control light emissions, the real impact of such frameworks is negligible.

Although ALAN is not commonly perceived as hazardous, due to lack of public awareness, its impact on the circadian rhythm, melatonin regulation, sleep and alertness and mood has been widely documented. Studies on night workers have been seminal in establishing ALAN as a lifestyle risk factor for sleep disorders, cancer, cardiovascular disease, type 2 diabetes, obesity, and depression, among many others. These studies have paved the way for researchers to conduct epidemiological studies in large cohorts using advanced satellite remote sensing technology to measure ALAN, aiming to corroborate the associations of ALAN with these diseases. Further translational and well-controlled studies in humans are needed to determine the causal effect of ALAN on health and to identify the underlying mechanisms.

While waiting for stricter regulation to mitigate light pollution, it is also our individual responsibility to take steps to reduce light pollution. For example, shielding all exterior lights to illuminate only what is intended, using shades to minimise interior light output, use of motion sensors or timers to automatically control exterior lighting, choosing warmer colours of light, use of dimmable lighting, and turning off the lights when not in use are some of the lifestyle changes that can be adopted at an individual level to limit the harmful impact of ALAN on health.

Although LEDs have contributed to tangible progress towards Sustainable Development Goal (SDG) 7 (ensuring affordable and clean energy for all) and SDG 8 (ensuring economic growth), this has been at the cost of impeding the progress towards achieving four other SDGs: SDG 3 (reducing the number of deaths and diseases caused by pollutants), SDG 13 (achieving synergies across climate action), SDG 14 (conserving life below water), and SDG 15 (conserving life on land). This unintentional trade-off of achieving one SDG at the cost of another calls for a wider re-evaluation of how SDG targets are achieved and their successes should not be measured in isolation.

It is ironic that while high-income and middle-income countries are suffering from ALAN, 675 million people worldwide did not have access to electricity in 2021, and according to the 2023 SDG report this figure is expected to remain at around 660 million in 2030. Compared to other environmental pollutants, ALAN as a pollutant, would be relatively easier to be regulated and mitigated if there is a universal intent and consensus.

